# A Rapid and Reliable Spectrofluorimetric Method to Measure the Urinary Lactulose/Mannitol Ratio for Dysbiosis Assessment

**DOI:** 10.3390/biomedicines12071557

**Published:** 2024-07-13

**Authors:** Lorenzo Marino Cerrato, Elisabetta Schiano, Fortuna Iannuzzo, Gian Carlo Tenore, Vincenzo Summa, Maria Daglia, Ettore Novellino, Mariano Stornaiuolo

**Affiliations:** 1 Department of Pharmacy, School of Medicine and Surgery, University of Napoli Federico II, Via Domenico Montesano 49, 80131 Napoli, Italy; lorenzomarinocerrato98@gmail.com (L.M.C.); giancarlo.tenore@unina.it (G.C.T.); vincenzo.summa@unina.it (V.S.); maria.daglia@unina.it (M.D.); 2 Inventia Biotech-Healthcare Food Research Center s.r.l., Strada Statale Sannitica KM 20.700, 81020 Caserta, Italy; elisabettaschiano@inventiabiotech.com (E.S.); ettore.novellino@unicatt.it (E.N.); 3 Department of Pharmacy, University of Chieti-Pescara G. D’Annunzio, 66100 Chieti, Italy; fortuna.iannuzzo@unich.it; 4 Department of Medicine and Surgery, Catholic University of the Sacred Heart, 00168 Rome, Italy

**Keywords:** LMR, spectrofluorimetry, urine, dysbiosis, mannitol, lactulose

## Abstract

Gut microbiota plays a crucial role in human health homeostasis, and the result of its alteration, known as dysbiosis, leads to several pathologies (e.g., inflammatory bowel disease, metabolic syndrome, and Crohn’s disease). Traditional methods used to assess dysbiosis include the dual sugar absorption test and the urinary lactulose/mannitol ratio (LMR) measurement using mass spectrometry. Despite its precision, this approach is costly and requires specialized equipment. Hence, we developed a rapid and reliable spectrofluorimetric method for measuring LMR in urine, offering a more accessible alternative. This spectrofluorimetric assay quantifies the fluorescence of nicotinamide adenine dinucleotide (NADH) and nicotinamide adenine dinucleotide phosphate (NADPH) produced during the enzymatic oxidation of mannitol and lactulose, respectively. The assay requires 100 µL of urine samples and detects LMR values lower (eubiosis) and higher (dysbiosis) than 0.05, ultimately being amenable to high-throughput screening and automatization, making it practical for clinical and research settings. A validation of the method demonstrated its high precision, accuracy, and robustness. Additionally, this study confirmed analyte stability under various storage conditions, ensuring reliable results even with delayed analysis. Overall, this spectrofluorimetric technique reduces costs, time, and the environmental impact associated with traditional mass spectrometry methods, making it a viable option for widespread use in the assessment of dysbiosis.

## 1. Introduction

Dysbiosis refers to a medical condition consisting of an alteration of the physiological balance (eubiosis) among families of commensal and symbiotic microorganisms (microbiota) normally resident in the gut of a healthy individual [1]. Dysbiotic conditions might manifest during pharmacological treatments, mostly with antibiotics (e.g., clarithromycin, metronidazole, and vancomycin) [1,2,3,4,5] and chemotherapy [3,6] or as a consequence of food intoxication [7], food allergy [8,9], and pathogen infections (e.g., *Clostridium*, *B. fragilis*, and *E. coli*) [1,4,5,10]. Interestingly, dysbiosis also manifests as a consequence of psychological stress (e.g., depression and anxiety) [11,12,13]. In its most severe manifestation, dysbiosis is prodromic to a wide range of human diseases, including dysmetabolism [1,10], inflammatory bowel disease (IBD), Crohn’s disease [1,10,14], colorectal cancer [15], metabolic syndrome [16], defects in iron absorption [4,17], and cachexia [8,11,14].

As a consequence of the strict connection linking intestinal cells’ health and gut microbiota, dysbiosis manifests as a loss of functionality of the intestinal barrier [16]. Indeed, the stress generated by a dysbiotic environment determines oxidative stress [11], inflammation [10], and leukocyte infiltration [14], ultimately leading to the loosening of claudin and cadherin junctions and the loss of the integrity of intestinal barrier function. These alterations all lead to increased intestinal permeability and, in the worst cases, the infiltration of bacteria and microbiota-derived metabolites into the bloodstream [16].

Due to the complexity and the large interindividual variation among human microbiota, dysbiosis is a condition difficult to diagnose. Notwithstanding, recent epidemiological evidence suggests dysbiosis affects millions of people worldwide [18].

Considering the high occurrence rate of dysbiosis in the general population, pharmaceutical and nutraceutical industries are investing in identifying drugs, nutraceuticals, and food supplements, as well as life habits, that might ameliorate dysbiosis or reduce its rate of occurrence [19].

The urinary lactulose to mannitol ratio (LMR) from a dual sugar absorption test is one of the biological parameters used for the diagnosis of dysbiosis and as a read-out of clinical trials aiming to the assessment of treatment efficacy [18,20]. Mannitol and lactulose are two inert sugars that are scarcely bioavailable and are passively absorbed from the intestine and excreted, nonmetabolized, within six hours from their oral intake in urine [20]. Around 10% of monosaccharide mannitol (MW = 182 g/mol) is absorbed transcellularly [21]. In eubiotic subjects, disaccharide lactulose (MW = 342 g/mol) is instead scarcely paracellularly absorbed (less than 1%) through tight junctions [21]. In dysbiotic conditions, due to increased gut permeability, lactulose is present in higher levels in urine, thus leading to increased LMR values [18].

The dual sugar absorption test used to measure the LMR in human urine samples is easy and non-invasive. Briefly, after the ingestion of the two sugars, patients are asked to collect urine for the following 6 h. In many protocols, the LMR is calculated after the ingestion of the two sugars in a 2:1 ratio (Sequeira et al. [20,21,22]). Although absolute LMR values for the assessment of dysbiosis have not been established yet, in eubiotic subjects, this urinary mass/mass ratio is generally lower than 0.05, with higher LMR values indicating altered intestinal permeability and dysbiosis [18].

So far, the technique used to assess the LMR has been mass spectrometry. Despite the fact that this approach is characterized by very low limits of detection (LODs) and limits of quantification (LOQs), this methodology requires expensive instruments and specific technical expertise.

Here, we developed an easy and suitable enzymatic fluorescence method for LMR measurement of a wide range of sugar concentrations, requiring only 110 µL of urine samples and being amenable to high throughput screening and automatization. Fluorescence assays would require more affordable instrumentation, as well as less specialized personnel [23]. In this assay, in a 96 multi-well plate, the urinary LMR is indirectly measured by quantifying the fluorescence emitted by nicotinamide adenine dinucleotide (NADH) and nicotinamide adenine dinucleotide phosphate (NADPH) produced during the enzymatic processing of mannitol and lactulose, respectively, by specific glycosidases [24]. In conclusion, our data suggest a new method that is especially useful for the analysis of a large set of samples in the clinical diagnosis of dysbiosis.

## 2. Materials and Methods

### 2.1. Reagents

D-Mannitol (purity ≥ 98% HPLC), lactulose (purity ≥ 98% HPLC), glacial acetic acid (CH_3_COOH), sodium hydroxide (NaOH), magnesium sulfate heptahydrate (MgO_4_S • 7 H_2_O), and hydrogen peroxide (H_2_O_2_) in a 30% solution (*w*/*w*) were purchased from Sigma-Aldrich (St. Louis, MO, USA). 2-Amino-2-(hydroxymethyl)-1,3 propanediol (Tris) (C_4_H_11_NO_3_) was purchased from Sigma-Aldrich (Mannheim, Germany). Urine samples, used as a matrix in our experiments, were obtained from unfiltered eubiotic gender-pooled human urine, commercially available from BioIVT (Burgess Hill, UK).

### 2.2. Rationale of Enzymatic Assays Determination

#### 2.2.1. Mannitol Enzymatic Assay Principle

Human urine contains more than 116 carbohydrates and conjugates of carbohydrates, with glucose being the most abundant (2 g/L) [25]. Despite the presence of D-arabitol and D-sorbitol, these do not interfere with urinary D-mannitol measurement; thus, it can be performed in a one-step reaction after oxidation to D-fructose by mannitol dehydrogenase (ManDH) in the presence of NAD^+^ with the formation of a stoichiometric amount of NADH.

#### 2.2.2. Mannitol Enzymatic Assay Principle

For lactulose measurement, urinary D-glucose (including urinary D-glucose generated by lactose hydrolysis) was a relevant interferent. Therefore, urine samples were pre-treated with a mixture of glycolytic enzymes that acted simultaneously to remove the interferent. Firstly, lactulose was hydrolyzed by the enzyme β-galactosidase to D-galactose and D-fructose. The enzyme β-galactosidase hydrolyzed lactose to D-galactose and D-glucose as well. The system was thus cleared from D-glucose interference by treatment with the enzymes glucose oxidase and catalase in the presence of O_2_. The D-fructose, generated from lactulose hydrolysis, was then phosphorylated by the enzyme hexokinase (HK) in the presence of adenosine-5′-triphosphate (ATP) to fructose-6-phosphate (F-6-P). F-6-P is converted to glucose-6-phosphate (G-6-P) by the enzyme glucose-6-phosphate isomerase (PGI). G-6-P was firstly oxidized to gluconate-6-phosphate (gluconate-6-P) by the enzyme glucose-6-phosphate dehydrogenase (G6P-DH) in the presence of NADP^+^ with the formation of reduced NADPH. Then, the produced gluconate-6-P was further oxidized to D-ribulose-5-phosphate and CO_2_ by the enzyme 6-phosphogluconate dehydrogenase (6-PGDH) in the presence of NADP^+^ with the formation of a second equivalent of reduced NADPH, with the amount of NADPH formed being stoichiometric to twice the amount of lactulose.

### 2.3. Spectrofluorimetric Method for Mannitol and Lactulose Measurements

Fluorescence measurements were performed using an EnVision™ Multimode Plate Reader (PerkinElmer 2105, Waltham, MA, USA). Samples were allocated in Corning Costar 96-well white polystyrene flat-bottomed plates (ref. 3654, Corning Inc., Acton, MA, USA). The instrument settings included the following: λ_exc_ = 320 ± 75 nm and λ_em_ = 480 ± 30 nm, the detector set to a height of 6.5 mm, detector gain at 100, and 10 flashes used for excitation. The difference in fluorescence emitted by samples (Δ_fluorescence_), before and after the addition of glycolytic enzyme and corrected from the autofluorescence of the blank, were graphed against metabolite concentration (mg/mL) to construct the calibration plot and obtain the correlated regression equation.

### 2.4. Assays Procedures

#### 2.4.1. Mannitol Assay Procedure

Reagents were adapted from Megazyme spectrophotometric kits (CAT. NO. K-MANOL and CAT. NO. K-LACTUL, Wicklow, Ireland) used for mannitol and lactulose measurements in food matrices. Briefly, for mannitol, 10 µL of the calibration standards, either dissolved in water or in urine, were added to 200 µL of water in the 96 multi-well plates, to be then supplemented with 20 µL of a solution containing NAD^+^ and a buffer at pH 9 in equal parts. Instead, 10 µL of water or urine were used for blank samples. After an initial fluorescence measurement, 2 µL of ManDH suspension were added to start the enzymatic reaction, reaching the final volume of 232 µL. Upon 4 min of incubation at RT, the fluorescence of samples was read and corrected via blank removal to finally obtain the value of Δ_fluorescence_.

#### 2.4.2. Lactulose Assay Procedure

For lactulose, 100 µL of calibration standards, dissolved in water or urine, were supplemented with 20 µL of a sodium acetate buffer 2M at a pH of 4.5 and 10 µL of β-galactosidase solution following the manufacturer’s instructions (for blank samples, the enzyme was replaced by 10 µL of water). When indicated, 100 µL of water or urine were used for blank samples. After vortexing for 1 min, the samples were incubated for 1 h at 40 °C in a water bath. Then, the samples were depleted of interferent sugars by a preclearing step, consisting of the addition of 100 µL of TRIS buffer 1M (pH 7.6) plus MgSO_4_ 10 mM, 10 µL of NaOH 0.33 M, and 10 µL of a mixture of the enzymes GOX/Catalase, being finally supplemented with 4 µL of H_2_O_2_ 30% (*w*/*w*). After mixing for 1 min, the samples were incubated for 15 min at 40 °C in a water bath and then centrifuged at 13,000 rpm for 10 min to remove urinary debris. In total, 200 µL of cleared supernatant were transferred in a 96 multi-well plate and supplemented with 10 µL of buffer at a pH of 7.6 and 10 µL of a solution of NADP^+^ and ATP. After mixing, 4 µL of a suspension containing the enzymes HK and G-6-PDH and 4 µL of a suspension of the enzyme 6-PGDH were added to start the enzymatic reactions. Upon 10 min of incubation at RT, the first fluorescence read was measured. Then, 4 µL of a suspension of the enzyme PGI were added to complete the enzymatic process. After mixing, and following 15 min of incubation at RT, the second fluorescence read was measured. Then, the Δ_fluorescence_, corrected by the removal of blank autofluorescence, was calculated.

### 2.5. Analytical Validation of the Spectrofluorimetric Method

#### 2.5.1. Linearity and Sensitivity of the Method

Method validation was performed according to the ICH validation guideline (ICH.Q2[R1], 1995) [26], which included the evaluation of a set of parameters, such as linearity range, the limit of detection (LOD), the limit of quantification (LOQ), precision, and accuracy. Stock solutions for mannitol and lactulose analytical standards were prepared by dissolving analytical powders in water to obtain 2 mg/mL and 1 mg/mL stock solutions, respectively. Dilutions were prepared from mother stocks, by diluting in water to reach final concentrations ranging from 1.5 to 0.008 mg/mL for mannitol (10-point calibration curve) and, for lactulose, from 0.25 to 0.0008 mg/mL (9-point calibration curve). The dilutions of both standards were analyzed in triplicate at each concentration. To evaluate the linearity of the standards response, the calibration curves (R^2^ ≥ 0.99) were constructed by plotting the intensity of the Δ_fluorescence_, corrected from the blank, of NADH and NADPH for mannitol and lactulose, respectively, against the standard concentrations. LOD and LOQ values were determined to evaluate the sensitivity of the method. Determination of the signal-to-noise ratio was performed by comparing measured signals from samples with low concentrations of the analyte with those of the blank samples. LOD represents the minimum concentration at which the analyte can be reliably detected and is defined as the lowest detectable concentration of the analyte that gives a response distinguishable from the background [S (signal of compound)/N (signal of noise) = 3.3]. LOQ is defined as the lowest quantifiable concentration of the analyte that can be measured with a standard level of confidence, and it is typically calculated using (S/N) = 10.

#### 2.5.2. Precision and Accuracy of the Method

According to the ICH validation guideline [26], the determination of accuracy (estimated by calculating the % bias) and precision (estimated by calculating the coefficient of variation CV %) was performed for the validation of the analytical method. Precision and accuracy parameters were calculated by intra-day and inter-day analysis at 3 different concentrations for both mannitol (1.5, 2.5, and 0.008 mg/mL) and lactulose (0.25, 0.0125, and 0.0008 mg/mL) standards. The intra-day analysis was performed by the reading of standards fluorescence 3 times per day, while the inter-day analysis was performed by the reading of standards fluorescence for 3 consecutive days.

### 2.6. Statistical Analysis

Samples were all measured in triplicates. Graphics were plotted using GraphPad 8.0 Prism software. The statistical analyses were performed using Microsoft^®^ Excel 2013 and GraphPad 8.0 Prism software. Unless otherwise stated, all the experimental results are expressed as the mean ± standard deviation (SD) of 3 repetitions, and the significance of the mean difference was evaluated by performing a *t*-test, applying Bonferroni–Dunn post hoc test as needed. Significant differences were accepted at the 95% confidence level, where *p* values of less than 0.05 were considered statistically significant.

## 3. Results and Discussion

### 3.1. Spectrofluorimetric Method Validation

The spectrofluorimetric method for the assessment of the urinary LMR in dysbiosis diagnosis was validated according to the ICH validation guideline. To this purpose, linearity, sensitivity, accuracy, and precision parameters were evaluated for both mannitol and lactulose. As described in Section 2.5.1*,* linearity studies were conducted by generating calibration curves on a wide range of analytical standard dilutions. All the analyses were performed in triplicate, yielding a linear relation, described by a correlation coefficient of R^2^ ≥ 0.99. The standard concentrations of mannitol and lactulose were plotted versus the Δ_fluorescence_ of NADH and NADPH, respectively. The sensitivity of the spectrofluorimetric method was assessed by determining LOD and LOQ values for both compounds. The linearity and sensitivity values of the method for both sugar compounds are reported in Table 1.

Since LOD and LOQ values for both sugars are largely below the concentrations detected and quantified in the dual absorption test reported in many studies [20,22], this analytical protocol can be considered a suitable and reliable method for LMR assessment.

Precision and accuracy were calculated for three different concentrations by an intra-day and an inter-day analysis of the assays performed for both mannitol (1.5–0.008 mg/mL) and lactulose (0.25–0.0008 mg/mL). As shown in Table 2, the % CV values for mannitol analysis ranged from 1.3 to 4.5% and 1.6 to 6.2% for intra-day and inter-day precision, respectively, while the % CV values for lactulose analysis ranged from 3.8 to 7.4% and 5.6 to 7.7% for intra-day and inter-day precision, respectively. Moreover, the % bias for mannitol analysis ranged from −2.1 to 1.5% and −2.1 to 4.0% for intra-day and inter-day accuracy, respectively, while the % bias for lactulose analysis ranged from −0.7 to −0.2% and −0.2 to 3.7% for intra-day and inter-day accuracy, respectively. According to the low values obtained for both % CV and % bias, the developed spectrofluorimetric method was demonstrated to be a reproducible and reliable protocol for the measurement of mannitol and lactulose.

### 3.2. Analytes Stability Study

**Figure 1:**
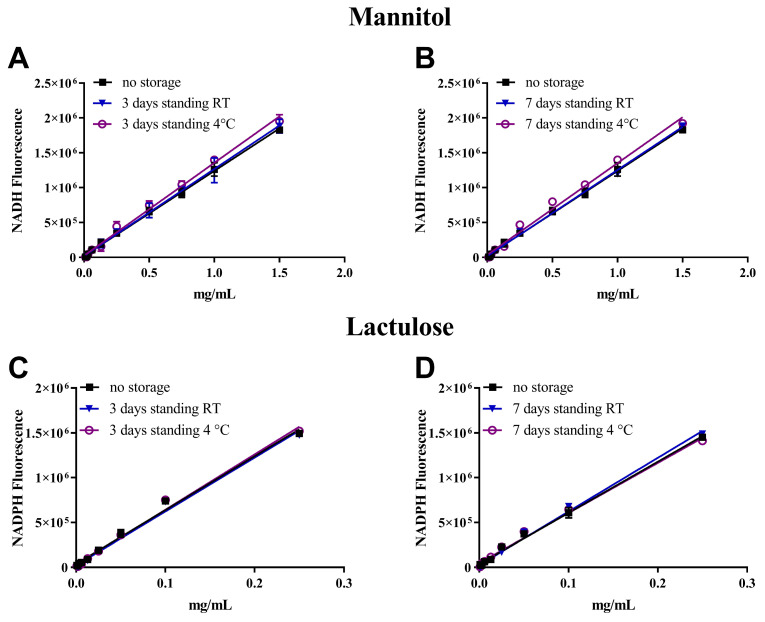
Calibration curves of mannitol (**A**,**B**) and lactulose (**C**,**D**) measured soon after dilution or stored at RT and 4 °C for 3 and 7 days. Dilutions were prepared from mother stocks to reach final concentrations ranging from 1.5 to 0.008 mg/mL for mannitol (10-points calibration curve) and, for lactulose, from 0.25 to 0.0008 mg/mL (9-points calibration curve). Values represent the average ± SD of three replicates. Statistical significance was calculated by Student’s *t*-test.

Analyte stability was evaluated for both sugars in two different storage conditions, i.e., RT and at 4 °C, for two time periods of 3 and 7 days. Multicentric clinical trials do not usually allow for the synchronous collection and analysis of the samples that are thus stored for multiple days in a fridge, freezer, or cooled boxes, before analysis. Interestingly, as can be noticed in Figure 1, no statistically significant differences were observed (*p* > 0.05) for both mannitol (A and B) and lactulose (C and D) measurements obtained from the assays performed in the different storage conditions tested compared to the assay performed on the freshly prepared dilutions.

### 3.3. Cross-Selectivity Assays and Urinary LMR Evaluation

According to the work of Sequeira et al. [22], 0.75 mg/mL represents the mean concentration of mannitol in both eubiotic and dysbiotic urinary samples collected during six hours from administration of the dual sugar absorption test. By contrast, the mean concentrations of lactulose in the urines of eubiotic and dysbiotic subjects are reported to be 0.075 mg/mL and 0.25 mg/mL, respectively. Firstly, we assessed the lack of cross-reactivity between the two fluorescence measurements, applying the fluorescence measurements to standard solutions, dissolved in water and urine, containing both lactulose and mannitol. No statistically significant differences (*p* > 0.05) were observed in the measurements of mannitol in samples containing eubiotic and dysbiotic amount of lactulose. Similarly, no statistically significant differences (*p* > 0.05) were observed in the measurements of eubiotic and dysbiotic lactulose in samples containing mannitol or not.

**Figure 2:**
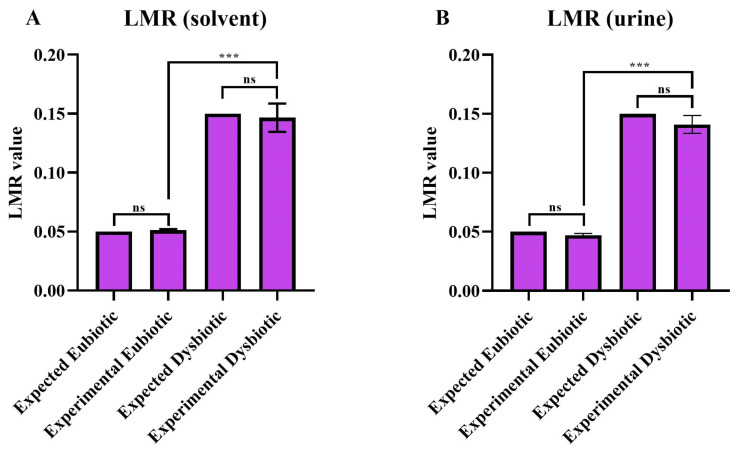
Comparison of LMR values calculated by fluorescence assay in dysbiotic and eubiotic samples. Fluorescent measurement in solvent (**A**) and urine (**B**) are then compared with their ab initio expected values. Values represent the average ± SD of three replicates. Statistical significance was calculated by Student’s *t*-test. *p* value *** < 0.0005; ns = non statistically significant.

Finally, LRM was measured in the urine samples according to the developed fluorescent assay. As shown in Figure 2A,B, the fluorescent assay provides a correct estimation of lactulose and mannitol concentration both in solvent and in human urine, thus allowing for a reliable identification of the phenotype of the patient.

## 4. Conclusions

The LMR is routinely measured by LC-MS analysis. However, the assessment of LMRs by mass spectrometry analysis is costly and time-intensive. Additionally, the substantial quantities of organic solvents required for HPLC or LC-MS analysis render these techniques environmentally unfriendly for routine medical diagnosis. Thus, there is an urgent need for a simple, green, swift, and cost-effective analytical method to determine LMRs in biological samples.

Spectrofluorimetric measurement emerges as a potent solution in analytical chemistry, offering high selectivity and sensitivity for detecting and quantifying fluorescent compounds, all without the need for complex equipment or the excessive use of organic solvents. In this current study, the oxidation of mannitol and lactulose in the urine of patients undergoing the two-sugar test produces stoichiometric amounts of NADH and NADPH, both exhibiting a native fluorescence band at 480 nm after excitation at 320 nm, which allowed for their determination on a small scale, using minimal amounts of non-hazardous solvents. We proved that the assay was amenable to discriminate among eubiotic (LMR < 0.05) and dysbiotic urinary samples (LMR > 0.05). We assessed the reliability of the procedure on samples stored at room temperature, as well as on samples stored for longer periods at different temperatures; while ideally, the sample should be immediately analyzed, we prove that storage at 4 °C for 3–7 days does not drastically compromise the accuracy of the assay and still allows for correct LMR estimation and dysbiosis diagnosis. This is particularly useful, considering the fact that clinical trials assessing dysbiosis are often multicentric and involve large cohorts of samples that are difficult to synchronize in terms of analysis.

Considering the reliability of the measurement, the LRM fluorescent assay here described offers several advantages over the traditional LC-MS technique approach. LRM fluorescence measurements require less preparation and time analysis compared to mass spectrometry methods, making our approach suitable for high-throughput analysis, which is beneficial when a large number of samples are simultaneously processed and analyzed.

Moreover, our assay is cost-effective and easy to use. This accessibility makes fluorescence assays more widely applicable, especially in settings where advanced expertise or sophisticated instrumentations may be lacking.

## Figures and Tables

**Table 1:** Linearity range, equation of calibration curves, linearity, limit of detection (LOD), and limit of quantification (LOQ) values of the spectrofluorimetric analysis for mannitol and lactulose.

Compound	Linearity Range (mg/mL)	Calibration Curve	R^2^	LOD (mg/mL) ^1^	LOQ (mg/mL) ^1^
Mannitol	0.008–1.5	y = 1,220,067x + 21,055	0.99	0.007 ± 0.001	0.022 ± 0.002
Lactulose	0.0008–0.25	y = 4,802,619x + 30,837	0.99	0.0006 ± 0.0001	0.0019 ± 0.0004

^1^ LOD and LOQ values represent the average ± standard deviation of three replicates.

**Table 2:** Precision (% CV) and accuracy (% bias) values of spectrofluorimetric intra-day and inter-day analysis for mannitol and lactulose by fluorescence measurements.

Compound	Concentration (mg/mL)	Precision (% CV)		Accuracy (% bias)	
		Intra-Day	Inter-Day	Intra-Day	Inter-Day
Mannitol	1.5	1.3	1.6	−1.9	2.8
	0.25	4.4	4.5	1.5	4.0
	0.008	4.5	6.2	−2.1	−2.1
Lactulose	0.25	7.4	7.5	−0.7	3.7
	0.0125	3.8	5.6	−0.4	−0.2
	0.0008	4.7	7.7	−0.2	−0.2

## Data Availability

Data are contained within the article.
